# Validity Analysis of Neck Circumference as a Screening Test for Hypoxia Occurrence in Patients Undergoing Sedative Endoscopy

**DOI:** 10.3390/healthcare10040679

**Published:** 2022-04-03

**Authors:** Hyun-Ji Song, Jiyun Kim

**Affiliations:** 1 Gastrointestinal Endoscopy Center, Gachon University Gill Medical Center, Incheon 21565, Korea; vision6694@gilhospital.com; 2 School of Nursing, Gachon University, Incheon 21936, Korea

**Keywords:** sedative endoscopy, hypoxia occurrence screening, neck circumference measurement

## Abstract

This study was performed to check the validity of and propose a cutoff point for measuring the neck circumference for screening hypoxia occurrence in patients undergoing sedative endoscopy. Data were collected from 168 patients who visited the Endoscopy Center of G University Hospital between 27 April 2020 and 12 June 2020 to undergo sedative endoscopy. Hypoxia occurrences were measured using sleep questionnaires (STOP-BANG and Berlin questionnaires), and the neck circumference measurements of the patients were compared. Neck circumference as a predictor of hypoxia and its sensitivity and specificity according to the cutoff values were high; thus, it is a valid screening test for hypoxia in patients undergoing sedative endoscopy. The most appropriate cutoff values for sitting neck circumference and lying neck circumference in men were 40.5 and 40.3, respectively, and those for women were 35.3 and 35.8, respectively. Hypoxia can be predicted in patients undergoing sedative endoscopy by measuring their neck circumference.

## 1. Introduction

When patients undergo endoscopy for the screening of gastric cancer or colon cancer, pain or discomfort may occur, and the patient’s involuntary movement or irregular breathing may affect the procedure [1]. Therefore, sedation is performed to enable an operator to conduct detailed observations and long-term treatment and to improve the accessibility of endoscopy by allowing patients to undergo examinations without pain or discomfort [2].

However, given that sedation has potential risks, such as hypoxia, it is essential to be prepared to prevent it [3]. The frequency of hypoxia in sedative endoscopy is approximately 6–18% depending on the drug and dose used, and serious cardiopulmonary complications are known to occur in approximately 1/1000 people [4]. When sedation is induced, the depth of sedation continuously changes because individual responses are diverse and difficult to predict. Furthermore, the margin of safety of sedative drugs is narrow, and complications should be prevented by evaluating the risk of hypoxia caused by airway obstruction, central respiratory depression, or sleep apnea [5,6]. 

Hypoxia caused by airway obstruction during sedation increases to 15% in patients with obstructive sleep apnea [7]. Therefore, it is necessary to develop a screening tool that can predict hypoxia due to respiratory failure or airway obstruction during sedative endoscopy.

The standard polysomnography test, which is the most objective and reliable diagnostic test for obstructive sleep apnea, takes a long time and is difficult to provide to everyone because of problems with cost [8]. To overcome these limitations, sleep survey questionnaires such as the STOP-BANG questionnaire, Epworth Sleepiness Scale, and the Berlin questionnaire, which are diagnostic tools based on various questionnaires, are widely used in clinical practice [9,10,11]. 

In the case of obstructive sleep apnea, airway obstruction easily occurs when sedative drugs are used, and this has been investigated as a possible cause of hypoxia [12]. Therefore, studies are needed to predict and prepare for hypoxia caused by airway obstruction due to obstructive sleep apnea, and patients undergoing sedative endoscopy should be thoroughly screened. Even before sedation, a simple and efficient hypoxia screening test is required. In the original study of the STOP-BANG questionnaire, the cut-off point of neck circumference was 43 cm for men and 41 cm for women [13]. In the sleep apnea study conducted on the Australian population, the cut-off point for neck circumference was 44 cm for men and 38 cm for women [14]. In a study conducted on 4581 Korean men and women, the neck circumference of sleep apnea patients was 39.55 cm, which was statistically significantly longer than that of the control group, which was 35.56 cm [8]. Regarding the use of a sleep questionnaire as a screening test, it can be difficult to accurately measure one’s sleeping habits, particularly when one is living alone. By contrast, neck circumference measurement is objective and simple; therefore, it has more utility in clinical practice. This study aimed to investigate the validity of neck circumference measurement by comparing its usefulness as a predictor of hypoxia in patients undergoing sedative endoscopy with sleep questionnaires, such as the STOP-BANG and Berlin questionnaires. This study aimed to evaluate the validity of neck circumference as a screening test for hypoxia occurrence in patients undergoing sedative endoscopy.

## 2. Materials and Methods

### 2.1. Study Design

This study aimed to determine the validity of neck circumference measurement for screening hypoxia occurrence in patients undergoing sedative endoscopy.

### 2.2. Research Environment and Participants

The subjects were aged 18 to 65 years old and visited the Endoscopy Center of G University Hospital between 27 April 2020 and 12 June 2020 for the sedative endoscopy examination. The following patients were excluded: those who were seriously ill with American Society of Anesthesiologists (ASA) class III or higher; had an allergy related to sedatives such as eggs, soybeans, and sulfite; underwent endoscopic procedures, such as endoscopic submucosal resection; or underwent colonoscopy only. Hypoxia was defined as a decrease of less than 90% in oxygen saturation [15]. After identifying the characteristics of the subject, the researcher asked for consent to participate in the study from subjects who met the subject’s inclusion criteria.

The number of subjects in this study was compared between the hypoxia occurrence and hypoxia-free groups in patients undergoing sedative endoscopy by using G*Power software developed by the Department of Experimental Psychology, Heinrich-Heine-University, Düsseldorf, Germany [16]. According to the comparison results of a previous study, the Berlin sleep questionnaire, and the STOP-BANG sleep questionnaire [15], a total of 161 subjects were investigated in both groups [15]. By using a power (1-β) and significance level of 0.8 and 0.05, respectively, a total of 169 people were selected. The dropout rate was approximately 5%, and one patient was excluded because of a soybean allergy. 

### 2.3. Research Tools

#### 2.3.1. STOP-BANG Sleep Questionnaire

The STOP-BANG questionnaire consists of 8 items on snoring, tiredness, observed apnea, blood pressure, body mass index (BMI) > 28 kg/m^2^, age ≥ 50, neck circumference (male ≥ 43 cm, female ≥ 38 cm), and gender. If a patient answered yes to more than five basic questions, the total score is five points; a patient with a score of five or higher is considered a high-risk patient for obstructive sleep apnea [17].

#### 2.3.2. Berlin Sleep Questionnaire

The Berlin questionnaire is divided into three categories, and patients with two or more positive categories are at high risk of sleep apnea [18]. The first category of the Berlin questionnaire consists of five questions about snoring and apnea during sleep; if the score was two points or more, the patient was judged positive. The second category comprises four questions about the degree of drowsiness and fatigue during the day; if the score is two or more, the patient is judged positive. The third category asks whether a patient had been diagnosed with hypertension or if the BMI was 30 kg/m² or more; if only one of them was positive, the patient was judged positive.

#### 2.3.3. Neck Circumference in a Sitting Position and a Lateral Decubitus Position

Neck circumference was measured in a sitting position based on a point perpendicular to the long axis of the neck at the height of the cricothyroid membrane; for endoscopy, neck circumference was measured twice and up to 0.1 cm with a tape measure while the subject was lying on a lateral decubitus position [15,19]. The neck circumference was measured after the nurse in the endoscopy room obtained consent from the subjects to participate in the study. In order to reduce the difference in the measured values between the measurers, only one nurse performed the measurement.

### 2.4. Ethical Considerations

This study was approved by the Institutional Review Board of G Hospital (IRB No. GDIRB2020-155). The researcher explained the contents of the study, the purpose of the study, the research procedure, and the ethical aspects such as anonymity and confidentiality to subjects who met the inclusion criteria. The subjects signed a written consent form after listening to the explanations.

### 2.5. Data Analysis

For the statistical processing of collected data, this study used the Statistical Package for the Social Sciences (SPSS) (SPSS Inc., Chicago, IL, USA) for Windows (version 25.0), and the statistical significance level was set at *p* < 0.05.

To determine the average difference and hypoxia occurrence based on the general characteristics of the subjects, χ² was performed using a t-test and cross-analysis. A t-test was conducted to compare the STOP-BANG and Berlin questionnaires and to obtain the mean and standard deviation of the neck circumference and hypoxia occurrence in the sitting and lying positions. To analyze the correlation between the decrease in oxygen saturation, the correlation coefficients of the size of the neck circumference in the sitting and lying positions were calculated, and the STOP-BANG and Berlin sleep questionnaires were used. The receiver operating characteristic (ROC) curve was used to compare the decrease in oxygen saturation and the performance of each test as a screening test for hypoxia, and the area under the ROC curve (AUC) was calculated to evaluate the accuracy of the diagnosis. In the case of neck circumference, the ROC curve was generated separately for men and women because the lengths of the hypoxia predictors were different.

## 3. Results

### 3.1. Hypoxia Occurrence According to Subject Characteristics, Sleep Questionnaire, and Neck Circumference

In this study, there were 23 and 145 patients with and without hypoxia, respectively. The hypoxia occurrence group had a mean age of 53.39 (±12.31; *p* = 0.187) and comprised 14 men and 9 women (*p* = 0.289). The average height, weight, and BMI of the subjects with hypoxia occurrence were 166.39 cm (± 7.69; *p* = 0.352), 78.38 (±14.88; *p* = 0.001), and 28.29 kg/m^2^ (±4.92; *p* <.001). There was no statistically significant difference between the hypoxia occurrence group and the hypoxia nonoccurrence group when the same conditions were applied by the doctor in charge of the sedatives (*p* = 0.465). There were 13 patients (56.5%) in the hypoxia occurrence group who underwent gastroscopy only, and a total of 10 patients (43.5%) in the hypoxia occurrence group underwent gastrointestinal endoscopy and gastroscopy at the same time (*p* = 0.801). Hypoxia occurred in 7 (30.4%) and 16 (69.6%) patients without and with a history of hypoxia, respectively (*p* = 0.032). 

According to the characteristics of the subjects, the items that had statistically significant differences with hypoxia occurrence were weight, BMI, ASA class, and history of hypoxia (*p* < 0.05). The items that had no significant difference from hypoxia were age, sex, height, drug (Propopol) dose, and test name (*p* > 0.05). 

In both the STOP-BANG and Berlin sleep questionnaires, the hypoxia group showed higher scores than the hypoxia non-existing group, and the length of the neck circumference measurement was longer in the hypoxia group than in the hypoxia non-existing group in both sitting and lying positions (Table 1).

### 3.2. Comparison of Performance as a Screening Test for Hypoxia

**Figure 1:**
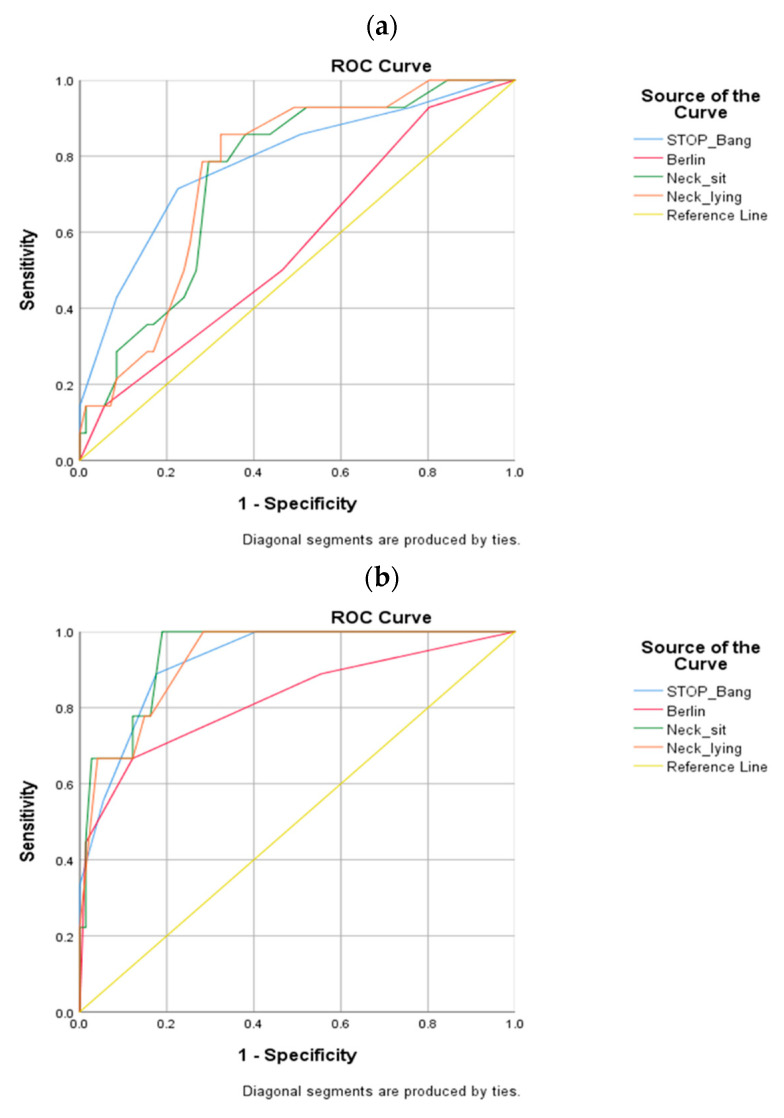
ROC curve by sex ((**a**) men and (**b**) women).

As a screening test for hypoxia, the ROC curve was used to compare the performance of the STOP-BANG and Berlin sleep questionnaires and the neck circumference length in the sitting and lying positions (Figure 1). The AUC was calculated to evaluate the accuracy of the diagnosis. In the case of neck circumference, the ROC curves were generated separately because the criterion length of the hypoxia predictors for men and women was different. 

The AUC values for the decrease in oxygen saturation by examination in men were as follows: STOP-BANG questionnaire, 0.785; lying position neck circumference, 0.759; sitting position neck circumference, 0.749; and Berlin questionnaire, 0.567. The AUC values of STOP-BANG were the highest. According to the AUC values, the STOP-BANG questionnaire and the numerical values of the neck circumference in the lying and sitting postures had moderate accuracy (0.7 < AUC ≤ 0.9), and the Berlin questionnaire had lower accuracy than the former tests (0.5 < AUC ≤ 0.7). 

The AUC values for the decrease in oxygen saturation by examination in women were 0.940 in the sitting position, 0.926 in the lying position, 0.923 in the STOP-BANG questionnaire, and 0.821 in the Berlin questionnaire. The AUC value of the neck circumference in the sitting position was the highest. The STOP-BANG questionnaire and the neck circumference in the lying and sitting positions were highly accurate (0.9 < AUC < 1) according to the AUC values, and the Berlin questionnaire was moderately accurate (0.7 < AUC ≤ 0.9) (Table 2).

### 3.3. Sensitivity, Specificity, and Cutoff Values for the Decrease in Oxygen Saturation for Each Test

To evaluate the accuracy of the STOP-BANG and Berlin questionnaires and the length of the neck circumference in the sitting and lying positions as a predictor of hypoxia according to the decrease in oxygen saturation, the sensitivity and specificity according to the cutoff value were identified and compared between male and female subjects (Table 3). The cutoff value with the highest sum of sensitivity and specificity was selected. To compare the cutoff value with the adjacent value, a higher value and a lower value than the cutoff value were inserted into the table, one each.

For men, when the STOP-BANG questionnaire score was 4.5 points, the sensitivity and specificity were 71% and 76%, respectively. When 0.5 categories of the Berlin questionnaire were positive, the sensitivity and specificity were 93% and 20%, respectively. When 1.5 categories of the Berlin questionnaire were positive, the sensitivity and specificity were 50% and 54%, respectively. When the neck circumference of the sitting position was 40.5 cm, the sensitivity and specificity were 79% and 70%, respectively. When the neck circumference in the lying position was 40.3 cm, the sensitivity and specificity were 86% and 68%, respectively.

For women, when the STOP-BANG questionnaire score was 2.5 points, the sensitivity and specificity were 89% and 82%, respectively. When 1.5 categories in the Berlin questionnaire were positive, the sensitivity and specificity were 67% and 88%. When the neck circumference of the sitting position was 35.3 cm, the sensitivity and specificity were 78% and 88%, respectively. When the neck circumference in the lying position was 35.8 cm, the sensitivity and specificity were 78% and 85%, respectively.

## 4. Discussion

The purpose of this study was to determine the validity of a neck circumference measurement for screening hypoxia occurrence during sedative endoscopy and to present it as a screening test for the prevention of hypoxia.

Among the general characteristics of the patients in this study, hypoxia occurrence had significant differences with BMI, ASA class, and history. Previous studies also reported old age, underlying disease, high ASA score, and obesity as risk factors for hypoxia, which is a complication of sedation endoscopy [20]. Obese patients have a high Mallampati score, which indicates very difficult airway intubation due to the structural deformation of the respiratory system, and they have a frequent occurrence of hypoxia [21]. In the current study, the average BMI of the hypoxia occurrence group was 28.29 kg/m^2^ (±4.92), which is considered obese. Patients with obesity have obstructive sleep apnea, which is presumed to be a result of the accumulation of fat in the upper respiratory tract wall or a decrease in upper respiratory function; in clinical practice, the drug dose and oxygen saturation in obese patients, respectively, increases and decreases owing to the frequent occurrence of sleep apnea [22,23]. Although obstructive sleep apnea improves in highly obese individuals after metabolic surgery, it is still necessary to maintain a normal weight to prevent hypoxia [24]. The other major factors of hypoxia include age and ASA class III or higher [20]. In the current study, there was no significant difference in age in patients aged 18 to 65 years old, excluding the elderly. ASA class II patients with underlying diseases such as hypertension or diabetes had higher hypoxia occurrences than ASA I patients without underlying diseases. Previous studies reported that hypoxia according to the drug and dose used at sedation was 6–18% [2,4,25]. In the current study, there was no statistically significant difference in the use of sedatives for moderate sedation under the order of an endoscopic doctor. However, according to the guidelines of the American Gastrointestinal Endoscopy Association, the European Gastrointestinal Endoscopy, and the Korean Gastrointestinal Endoscopy, medical staff who is responsible for sedation in sedative endoscopy should conduct a separate monitoring process [26] because it is believed that hypoxia due to sedative drugs can be prevented if a nurse in charge of sedative drugs provides professional monitoring and hypoxia prevention and treatment. 

The STOP-BANG, which is a screening tool for obstructive sleep apnea; the Berlin sleep questionnaire; and neck circumference were found to have a statistically significant difference with hypoxia occurrence at less than the significance level (*p* < 0.05), thus showing that all tests can be used as a screening tool for hypoxia [8,27,28].

Regarding the use of the STOP-BANG and Berlin questionnaires as screening tests for hypoxia around the neck in sitting and lying positions, the AUC value was calculated to confirm the usefulness of each test for reducing oxygen saturation. This is the same result as when considering the usefulness of the questionnaire for the diagnosis of obstructive sleep apnea. The AUC of the Berlin test remained in the 0.5-point range; therefore, the diagnostic usefulness of the Berlin questionnaire was not very high [18]. The AUC values for each test for the decrease in oxygen saturation in women were 0.940 for neck circumference in the sitting position, 0.926 for neck circumference in the lying position, 0.923 in the STOP-BANG questionnaire, and 0.821 in the Berlin questionnaire, thus showing that the neck circumference in sitting and lying positions and the STOP-BANG test were very accurate (0.9 < AUC ≤ 1) and that the Berlin test had moderate accuracy (0.7 < AUC ≤ 0.9). This suggests that the Berlin test is not a highly useful tool for screening hypoxia in patients undergoing sedative endoscopy. For women, the AUC value of the neck circumference in the sitting position was the highest, and the neck circumference measurement was more accurate than the sleep questionnaires.

Regarding the Berlin questionnaire, there is a possibility that sleep status due to insomnia or fatigue due to diseases, such as cancer, may have increased the sleep score regardless of sleep apnea; therefore, the Berlin questionnaire had an effect as a screening tool [29,30]. When surveying the sleep questionnaire in the current study, there were many cases where subjects were reluctant to talk about snoring or were not sure of their actual snoring state. On the other hand, neck circumference is an objective variable, and previous studies have shown that neck circumference is a useful and important predictor of sleep apnea [8,30,31]. In the current study, it was found that neck circumference is a valid predictive test for hypoxia in endoscopy patients during sedation. In clinical practice, where it is cumbersome to measure the neck circumference in sitting and lying positions, the sensitivity of the neck circumference in the lying position in men was found to be higher than that in the sitting position; for women, because the sensitivity is the same, it would be better to measure the neck circumference in a lying position with high sensitivity to increase the detection accuracy of hypoxia.

This study has some limitations. First, the results of this study were obtained from a limited number of patients who were 18–65 years of age with ASA I–II, excluding the elderly with high-risk diseases. Second, owing to the small number of sample groups, it is difficult to generalize the results, and further studies are required. Third, for patients who have undergone sedative endoscopy, the procedure is mixed as screening and therapeutic procedures for the purpose of visiting the hospital. In future studies, it is necessary to repeat the study with homogeneous subjects for the same purpose of endoscopy.

## 5. Conclusions

The STOP-BANG questionnaire predicted hypoxia more accurately than the Berlin questionnaire because neck circumference was included in the measurement. Neck circumference and the STOP-BANG test were useful predictors of hypoxia in both men and women undergoing sedation endoscopy. As for the sensitivity and specificity of the decrease in oxygen saturation, the neck circumference measurement showed higher sensitivity and specificity at the cutoff value than the STOP-BANG test.

Compared to the STOP-BANG questionnaire, measuring only the neck circumference is an objective measurement and is convenient, so it is useful to use the neck circumference as a screening test for hypoxia in patients undergoing endoscopy.

## Figures and Tables

**Table 1:** General characteristics, sleep questionnaire, and neck circumference classified with hypoxia occurrence.

Variable	Category	No Hypoxia (n = 145) Mean (SD)	Hypoxia (n = 23) Mean (SD)	*t*/*χ^2^*	*p*
Age (year)		49.70 ± 12.45	53.39 ± 12.31	−1.325	0.187
Sex	Man	71 (49.0)	14 (60.9)	1.125	0.289
	Woman	74 (51.0)	9 (39.1)		
Height (cm)		164.70 ± 8.16	166.39 ± 7.65	−0.933	0.352
Weight (kg)		65.76 ± 14.10	78.38 ± 14.88	−3.806	0.001
BMI (kg/m^2^)		24.10 ± 3.91	28.29 ± 4.92	−3.900	0.001
Propofol amount (mg)		3.10 ± 1.17	2.88 ± 1.26	0.732	0.465
ASA class	Status I	80 (47.3)	6 (3.6)	6.403	0.011
	Status II	66 (39.1)	17 (10.1)		
Examination	Gastroscopy only	86 (59.3)	13 (56.5)	0.064	0.801
	Gastroscopy and Colonoscopy	59 (40.7)	10 (43.5)		
History	No	79 (54.5)	7 (30.4)	4.595	0.032
	Yes	66 (45.5)	16 (69.6)		
STOP-BANG		2.48 ± 1.603	4.74 ± 1.514	6.330	<0.001
Berlin		1 ± 0.847	1.74 ± 0.964	3.817	<0.001
Neck-sit (cm)		35.71 ± 4.00	40.35 ± 4.06	5.158	<0.001
Neck-lying (cm)		36.43 ± 4.10	41.20 ± 4.22	−5.154	<0.001

**Table 2:** Comparison of AUC in the sleep questionnaire and neck circumferences for hypoxia due to sedative endoscopy.

	Variable	AUC ^†^	Standard Error	95% Confidence Interval
Men	STOP-BANG	0.785	0.072	0.643–0.926
	Berlin	0.567	0.082	0.406–0.728
	Neck-sit	0.749	0.064	0.624–0.875
	Neck-lying	0.759	0.061	0.639–0.878
Women	STOP-BANG	0.923	0.037	0.851–0.996
	Berlin	0.821	0.088	0.647–0.994
	Neck-sit	0.940	0.030	0.872–0.998
	Neck-lying	0.926	0.035	0.857–0.996

^†^ AUC: area under the curve.

**Table 3:** Sensitivity and specificity of oxygen saturation in men’s sleep apnea screening tests.

Sex	Variable	Cutoff	Sensitivity	Specificity	Sensitivity + Specificity
Men	STOP-BANG	3.5	0.857	0.493	1.350
		4.5	0.714	0.775	1.489
		5.5	0.429	0.915	1.344
	Berlin	0.5	0.929	0.197	1.126
		1.5	0.500	0.535	1.035
		2.5	0.143	0.944	1.087
	Neck-sit	39.8	0.786	0.662	1.448
		40.5	0.786	0.704	1.490
		41.3	0.500	0.732	1.232
	Neck-lying	39.8	0.857	0.634	1.491
		40.3	0.857	0.676	1.533
		40.8	0.786	0.676	1.462
Women	STOP-BANG	1.5	1.000	0.595	1.595
		2.5	0.889	0.824	1.713
		3.5	0.556	0.946	1.502
	Berlin	0.5	0.889	0.446	1.335
		1.5	0.667	0.878	1.545
		2.5	0.444	0.986	1.431
	Neck-sit	34.9	0.778	0.851	1.629
		35.3	0.778	0.878	1.656
		35.8	0.667	0.878	1.545
	Neck-lying	35.3	0.778	0.838	1.616
		35.8	0.778	0.851	1.629
		36.3	0.667	0.878	1.545

## Data Availability

The data that support the findings of this study are available from the corresponding author, upon reasonable request.
